# Antimicrobial resistance surveillance in Europe and beyond

**DOI:** 10.2807/1560-7917.ES.2018.23.42.1800560

**Published:** 2018-10-18

**Authors:** Gunnar Skov Simonsen

**Affiliations:** 1 Norwegian Organization for Surveillance of Antimicrobial Resistance (NORM), University Hospital of North Norway, Tromsø, Norway; 2 Faculty of Health Sciences, UiT – The Arctic University of Norway, Tromsø, Norway

**Keywords:** antimicrobial resistance, surveillance, public health, MRSA, GLASS, Kor-GLASS, Korea

Antimicrobial resistance (AMR) is a serious threat to global public health [1], but its clinical impact is difficult to determine at a population level. The mortality of systemic infections with extended-spectrum beta-lactamases (ESBL) Enterobacteriaceae and meticillin-resistant *Staphylococcus aureus* (MRSA) is approximately double that of infections by susceptible pathogens, even in modern healthcare systems [2]. The deleterious effects of AMR in low- and middle-income countries are largely unknown.

National, regional and global action plans all point to surveillance as a crucial first step to control and prevent AMR. Surveillance data are needed to estimate the scope of the problem, guide empirical treatment, monitor trends, detect new resistance phenotypes and measure the effect of interventions. However, no single surveillance scheme can achieve all of these goals, and the optimal design of such programs has not yet been established. In 2015, the World Health Organization (WHO) presented a global action plan on AMR and launched the Global antimicrobial resistance surveillance system (GLASS) as a key component to strengthen the evidence base for further activities [1,3]. During the early implementation phase, from 2015–19, countries are invited to register for participation and submit aggregated resistance data on human priority bacterial pathogens from relevant clinical specimens. Surveillance is organised at defined sentinel sites, where microbiological information is supplemented by epidemiological data on outpatient and inpatient numbers, as well as the numbers of patients with positive and negative cultures per specimen type and with susceptible and non-susceptible pathogens for each priority pathogen, stratified according to key demographic parameters.

This issue of *Eurosurveillance* contains two papers on the establishment of the national Kor-GLASS surveillance programme in South Korea. The first paper describes the organisation, structure and costs of the programme [4], whereas the second presents the results from the first 1-year report covering May 2016 to April 2017 [5]. Kor-GLASS is closely aligned with the GLASS protocol and based on the principles of representativeness, specialisation, harmonisation and localisation. Six sentinel hospitals with outpatient clinics and between 655 and 1,000 beds were recruited in four of the country’s nine regions, based on specific requirements for IT infrastructure and quality of microbiological laboratory services. The GLASS protocol was supplemented with additional national priority organisms and antimicrobial agents. All isolates were transferred to a central analysis centre for harmonised verification of species identification, phenotypic susceptibility testing, detailed molecular characterisation and storage. Clinical information was retrieved by laboratory personnel and submitted to the analysis centre. The overall cost of EUR 823,077 equalled, on average, EUR 74.20 for each of the 11,091 isolates included in the first year of the programme. Additional surveillance activities were established to collect *Neisseria gonorrhoea* and *Shigella* spp., as these species were not readily available at the sentinel hospitals. The first year results demonstrated high rates of multidrug resistance in organisms from all specimen types. Among blood culture isolates, there was 54.3% MRSA among all *Staphylococcus aureus*, 29.0% vancomycin resistance in *Enterococcus faecium*, 34.7% cefotaxime resistance in *Escherischia coli* (mostly ESBL) and 27.0% cefotaxime resistance in *Klebsiella pneumoniae*. Resistance rates were higher in infections acquired in hospitals than in the community, and the highest rates were found in intensive care units. The prevalence of bloodstream infections (BSI) with major AMR pathogens among inpatients was calculated for comparison with foreign countries. The most prevalent mutidrug-resistant phenotypes were cefotaxime resistant *E. coli* (2.1 BSI/10,000 patient days), MRSA (1.6 BSI/10,000 patient days), imipenem resistant *Acinetobacter baumannii* (1.1 BSI/10,000 patient days) and cefotaxime resistant *K. pneumoniae* (0.8 BSI/10,000 patient days). The authors conclude that the results of Kor-GLASS helped plan national action in response to the high rates of drug resistance.

AMR surveillance at the European level is presently organised through the European Antimicrobial Resistance Surveillance Network (EARS-Net) [6] and the Central Asian and Eastern European Surveillance of Antimicrobial Resistance (CAESAR) [7]. EARS-Net covers European Union and European Economic Area countries, whereas CAESAR includes all remaining countries in the WHO European Region. The two systems use similar surveillance strategies and protocols, taking into account differences in availability of resources. There is close collaboration between EARS-Net, CAESAR and GLASS, with European surveillance data routinely being transferred to the GLASS database. However, there are major differences between the GLASS protocol and the methodology used in Europe.

EARS-Net and CAESAR are laboratory-based surveillance programs focused on the collection of routine antimicrobial susceptibility data. EARS-Net includes data generated in ca 1,080 clinical laboratories that serve more than 1,600 hospitals across the continent. No centralised microbiological analyses are performed, but the programme is supported by external quality assessment and tailored tools for data management [6]. Denominator data are limited to estimate the coverage and representativeness of population, hospitals and isolates, and they therefore cannot be readily used for calculating the public health burden of AMR. Furthermore, EARS-Net and CAESAR are restricted to isolates from blood cultures and cerebrospinal fluids because of the heterogeneity in microbiological sampling practices for non-systemic infections. The strength of the European approach is the utilisation of vast amounts of readily available resistance data at limited cost.

The South Korean example holds important experiences and lessons for the future development of AMR surveillance in Europe and across the world. South Korea should be commended for its effort to establish a high-quality programme with a strong focus on harmonised and detailed microbiological analyses, as well as collection of relevant clinical data. The results presented in this issue of *Eurosurveillance* and in future publications will inform AMR containment activities at local and national levels. However, there are also limitations with the Korean model. The epidemiological validity of the data depends on the representativeness of sentinel hospitals as sampling sites within the South Korean healthcare system. The recruitment of relatively large facilities with access to adequate laboratory resources may skew the results in unpredictable ways. The collection of clinical data are described as ‘very burdensome’ for laboratory personnel, thus calling into question the sustainability of this part of the protocol. Finally, the considerable running costs may prove prohibitive, especially in low- and middle-income countries. Even with strong political and financial support, high running costs will necessarily limit the overall coverage of the system.

International AMR surveillance must build on national structures and systems that are adapted to specific features and needs of individual countries. The quest for comparability of data at regional and global levels must therefore be reconciled with the applicability of protocols and utility of data at the national level. In addition, AMR surveillance systems need to be designed according to the questions they are supposed to answer. Both EARS-Net/CAESAR and GLASS focus on the overall epidemiology of susceptible and resistant bacteria; however, they are not geared towards rapid alerts on unusual events, such as the discovery of new resistance phenotypes or emerging spread to new locations. Separate early warning systems have therefore been established at both the European Centre for Disease Prevention and Control (ECDC) and the WHO, but it is fair to say that these mechanisms are still in their early stages. Finally, there is a need to debate what the targets of surveillance programs should be and what should be answered by specific projects, using rigorous scientific methodology. It is tempting to suggest ongoing routine surveillance as the answer to any relevant question, but this is both scientifically flawed and financially unsustainable. The revolution in DNA sequencing technology has paved the way for detailed surveys of clone emergence, spread and decay, and in-depth analyses of patient determinants and outcomes can inform us in ways that routine surveillance can never do. ECDC has therefore supported targeted AMR projects outside the routine surveillance scheme [8,9]. The optimal design and uptake of scientifically valid and cost-effective AMR surveillance programs should be the topic of future research in different epidemiological settings and societies. A call for this was recently announced by the Joint Programming Initiative on Antimicrobial Resistance (JPI-AMR) [10].
